# Advancement of the 10-species subgingival Zurich Biofilm model by examining different nutritional conditions and defining the structure of the *in vitro* biofilms

**DOI:** 10.1186/1471-2180-12-227

**Published:** 2012-10-05

**Authors:** Thomas W Ammann, Rudolf Gmür, Thomas Thurnheer

**Affiliations:** 1 Section of Oral Microbiology and Immunology, Institute of Oral Biology, Center of Dental Medicine, Plattenstrasse 11, 8032, Zürich, Switzerland

**Keywords:** Periodontitis, Model, Biofilm, Subgingival, Structure, CLSM, FISH, IF

## Abstract

**Background:**

Periodontitis is caused by a highly complex consortium of bacteria that establishes as biofilms in subgingival pockets. It is a disease that occurs worldwide and its consequences are a major health concern. Investigations *in situ* are not possible and the bacterial community varies greatly between patients and even within different loci. Due to the high complexity of the consortium and the availability of samples, a clear definition of the pathogenic bacteria and their mechanisms of pathogenicity are still not available. In the current study we addressed the need of a defined model system by advancing our previously described subgingival biofilm model towards a bacterial composition that reflects the one observed in diseased sites of patients and analysed the structure of these biofilms.

**Results:**

We further developed the growth media by systematic variation of key components resulting in improved stability and the firm establishment of spirochetes in the 10-species subgingival Zurich biofilm model. A high concentration of heat-inactivated human serum allowed the best proliferation of the used species. Therefore we further investigated these biofilms by analysing their structure by confocal laser scanning microscopy following fluorescence *in situ* hybridisation. The species showed mutual interactions as expected from other studies. The abundances of all organisms present in this model were determined by microscopic counting following species-specific identification by both fluorescence *in situ* hybridisation and immunofluorescence. The newly integrated treponemes were the most abundant organisms.

**Conclusions:**

The use of 50% of heat-inactivated human serum used in the improved growth medium resulted in significantly thicker and more stable biofilms, and the quantitative representation of the used species represents the *in vivo* community of periodontitis patients much closer than in biofilms grown in the two media with less or no human serum. The appearance of *T. denticola, P. gingivalis*, and *T. forsythia* in the top layer of the biofilms, and the high abundance of *T. denticola,* reflects well the microbial situation observed at diseased sites. The improved model biofilms will allow further investigations of interactions between individual species and of the effects of atmospheric or nutritional changes, as well as interactions with tissue cells.

## Background

In the oral cavity, bacteria encounter many different stress factors. Shear-forces and high flow rates of saliva dominate on exposed surfaces, while bacteria colonizing the gingival crevices and/or subgingival pockets have to contend and withstand with the host’s immune response. As in most other environments, bacteria form biofilms as protection from these harsh conditions [1].

The bacterial community colonizing the oral cavity is highly complex and varies considerably between different individuals. According to current reports, 600 to 700 established species and likely several thousand only partially cultivable taxa can be detected [2]. However, this consortium does not pose a threat to a healthy individual. It even has a protective function by preventing the establishment or predominance of harmful organisms [3]. Several factors like imbalanced nutrition, smoking, diabetes, emotional stress, or genetic predisposition [4] can lead to changes in the composition of this subgingival community, leading to a loss of the natural ecological balance. Potentially pathogenic species may increase in numbers, starting to cause persistent infections of host tissues that are capable to cause not only tooth loss and bone resorption but also can spread out to extra-oral sites and become systemic [5]. Physical removal of the biofilms, possibly followed by an antibiotic treatment in refractory cases, is used to re-establish a biofilm community compatible with clinical stability. However, treatment will never and should not remove all organisms, since this could lead to settlement of even more harmful organisms. It is an almost impossible task to identify and selectively target only the actual pathogens among the hundreds of different species present [6].

Out of the potentially thousands of species found in the oral cavity, about 400 can be detected in periodontal pockets. This number is reduced to a range of 100 to 200 species in one patient [7]. The enormous diversity makes subgingival biofilms difficult to study and it seems impossible to fully understand all the interactions between the species. To investigate and better understand the role of individual species, models reflecting subgingival colonization are needed. Regarding the sophisticated structure of these biofilms [8], it is obvious that biofilms consisting of only one or two organisms do not sufficiently mirror the *in vivo* situation. Some investigators solved this problem by using inocula taken from diseased sites of patients [9,10]. Major problems in such model systems are both the restricted possibilities for analysis of all species involved and the composition of the inoculum, which inevitably varies substantially between donor patients.

An *in vitro* model system for subgingival biofilms should not only be functional in terms of pathogenic potential, it should also have a defined structure and a quantitative relationship between the species that resemble to some extent the *in vivo* situation. The aim of this study was therefore to further develop our 10-species model system [11] by 1) incorporating treponemes and balancing the growth medium to optimize their growth and 2) defining the structure of the produced biofilms. The incorporation of *Treponema denticola*, replacing *Treponema lecithinolyticum* used in our previous study, along with the variation of the growth medium allowed the treponemes to firmly establish in the biofilms. Further, *F. nucleatum* subsp. *vincentii* KP-F2 (OMZ 596), *Campylobacter rectus* (OMZ 697), *Streptococcus intermedius* ATCC 27335 (OMZ 512) were replaced by better growing strains (see methods).

The described modified model provides the possibility to examine the impact of variable growth conditions as well as the role of individual species. The high complexity of our 10-species model provides biofilms that are much closer to the *in vivo* situation than other models using just one or two species.

## Results

### Development of biofilms

Three different growth media were compared regarding bacterial abundances and biofilm stability: **SAL** (60% pooled, heat inactivated saliva, 30% modified fluid universal medium containing 0.3% Glucose [mFUM; [12]] and 10% heat-inactivated human serum), **mFUM4** (100% mFUM containing 4 mM glucose), and **iHS** (50% heat-inactivated human serum and 50% mFUM with 4 mM glucose. They all had low final concentrations of glucose: 4 mM for iHS and mFUM4, and 5.5 mM for SAL respectively. The formation of the biofilms was observed by determination of total counts on Columbia blood agar (CBA) plates at 5 time points during the incubation time. The final structure, as well as the thickness of the biofilms at 5 time points during the incubation time, was determined by confocal laser scanning microscopy (CLSM). The experiments confirmed and extended our previous finding [11] that the composition of the growth medium has a major effect on the development, stability and composition of the biofilms.

**Figure 1:**
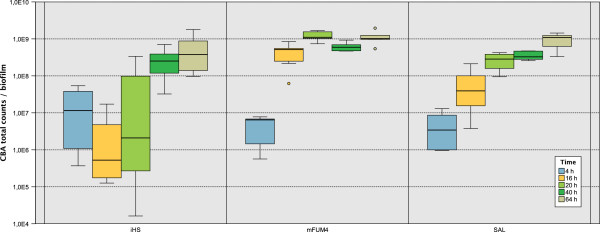
**Time course of biofilm growth comparing SAL, mFUM4, and iHS as growth media.** Total counts determined by plating on CBA agar plates (*T. denticola* and *T. forsythia* are not cultivable on CBA). Each box represents N = 9 independent biofilms from three independent triplicate experiments. The boxes represent the inter quartile range of the data points, the bar indicates the median. The whiskers cover the data points within the 1.5x inter quartile range. Dots are outliers within 1.5 and 3 box lengths outside the interquartile range.

**Figure 2:**
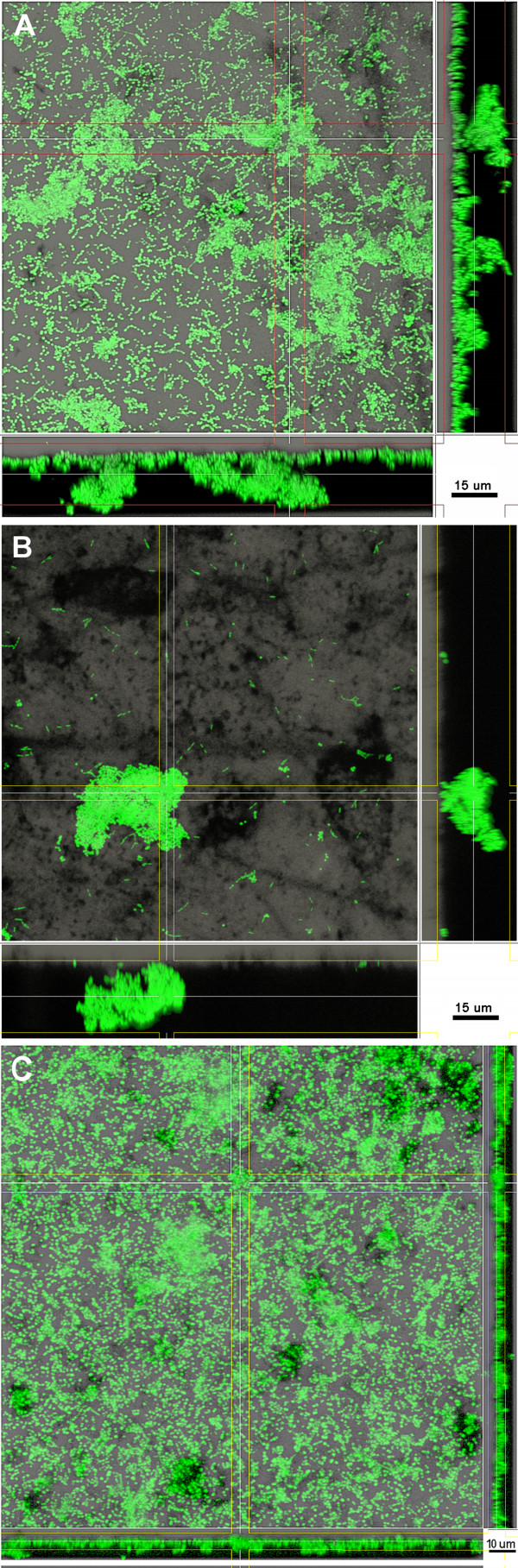
**Bacterial attachment to the disc surface under different nutritional conditions 4 h after inoculation.** Comparison of the growth media mFUM4 (**A**), iHS (**B**) and SAL (**C**). green: DNA staining using YoPro-1 + Sytox. The disc surface is visualized in grey colour. The images show representative areas of one disc each. Scale bars: 15 μm (A/B) and 10 μm (C).

The iHS medium delayed biofilm formation by 20 h compared to mFUM4 (Figure 1). 4 h after inoculation in mFUM4, the discs were densely colonized by cocci. Based on the observation that most of these cocci appeared as chains, they can be assumed to be streptococci. However, after 4 h of incubation in iHS, cocci were observed to appear almost exclusively as dense microcolonies, while rods (morphologically *Fusobacterium nucleatum*, *Prevotella intermedia, or Tannerella forsythia*) in low abundance colonized the majority of the disc. Incubation in SAL medium led to a similar observation as in mFUM4: The disc was colonized mainly by cocci (Figure 2).

**Figure 3:**
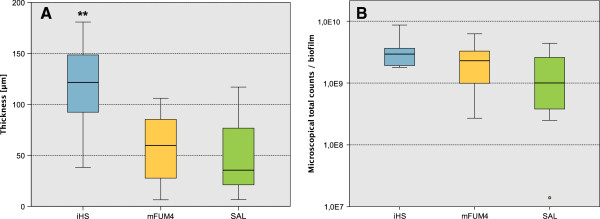
**Thickness (A) and total counts (FISH/IF) (B) of biofilms grown for 64.5 h in SAL, mFUM4, and iHS growth medium.** Thickness was determined by CLSM, total counts were calculated from the species specific quantification by visual microscopic counting following FISH- or IF from N=9 independent biofilms from three independent experiments. Thickness was determined at N=44 (iHS), N=61 (mFUM4) and N=57 (SAL) randomly selected measurement spots on the discs. The boxes represent the inter quartile range of the data points, the bar indicates the median. The whiskers cover the data points within the 1.5x inter quartile range. Dots are outliers within 1.5 and 3 box lengths outside the interquartile range. ** indicates the significantly higher thickness (p≤0.001) of iHS biofilms compared to biofilms of both SAL and mFUM4.

The high concentration of human serum in iHS improved biofilm stability in terms of firm attachment to the disc (less cell loss during dip washing and the FISH staining procedure), and further the average thickness of the biofilms was significantly increased after 64.5 h when compared to biofilms grown in mFUM4, or SAL respectively (Figure 3A). However, the total counts of bacteria per biofilm did not show significant differences between the three growth media (Figure 3B). 

In mFUM4, biofilms showed a rapid increase in biofilm thickness and total counts right after inoculation and reached their highest cell numbers after 20 h. While stable until then, they tended to partially detach from the discs during the dip-washes at later time points. In contrast, major parts of biofilms grown in iHS detached during the dip-washes in the first 20 h of incubation. This observation is in accordance with the strong decrease in total counts along with a high variability between different experiments and replicates. During further incubation, however, the remaining parts had stabilized and the biofilms showed a rapid increase in thickness and total counts. Biofilms cultivated in SAL medium showed a constant increase of total counts and thickness and were not prone to detachment during the incubation time (Figure 1).

### Quantitative representation of species in biofilms

**Figure 4:**
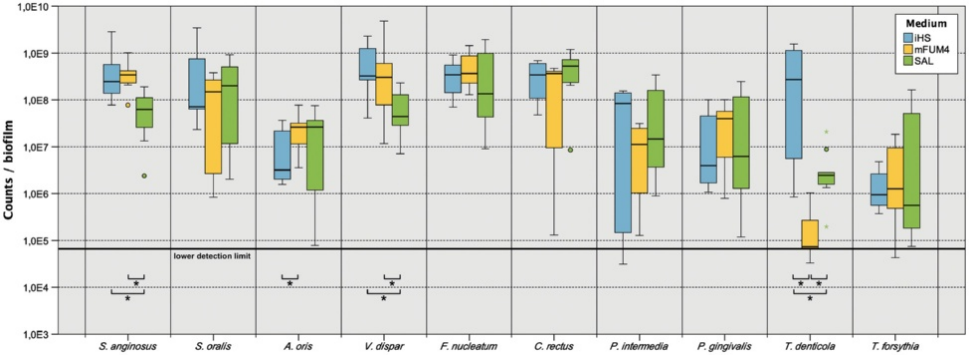
**Quantification of bacteria in biofilms grown for 64.5 h in SAL, mFUM4, and iHS growth medium.** Bacteria were quantified by visual microscopic counting. Each box represents N=9 independent biofilms from three independent experiments. The boxes represent the inter quartile range of the data points, the bar indicates the median. The whiskers cover the data points within the 1.5x inter quartile range. Dots are outliers within 1.5 and 3 box lengths outside the interquartile range, and colored stars are extremes that are more than 3 boxlengths outside the interquartile range. * indicate significant differences with p≤0.05 between a pair of boxes, as indicated by the brackets.

We determined the cell numbers of all organisms in biofilms grown either in SAL, mFUM4, and iHS medium. Enumeration of cells was performed by microscopical counting following staining the bacteria by fluorescence *in situ* hybridisation (FISH) or immunofluorescence (IF). The data are summarized in Figure 4. *Treponema denticola* showed significantly higher cell numbers in iHS compared to SAL and mFUM4 and was among the most abundant organisms in the biofilm. In mFUM4, *Treponema denticola* hardly proliferated and only appeared in abundances close to the detection limit. *Streptococcus anginosus* and *Veillonella dispar* showed significantly reduced growth in SAL medium compared to the other two media, while *Actinomyces oris* showed significantly reduced growth in iHS compared to mFUM4.

The abundances of *Streptococcus oralis*, *F. nucleatum*, *Campylobacter rectus*, *P. intermedia*, *Porphyromonas gingivalis*, and *T. forsythia* were not affected by the growth medium.

### Structure of mature biofilms

**Figure 5:**
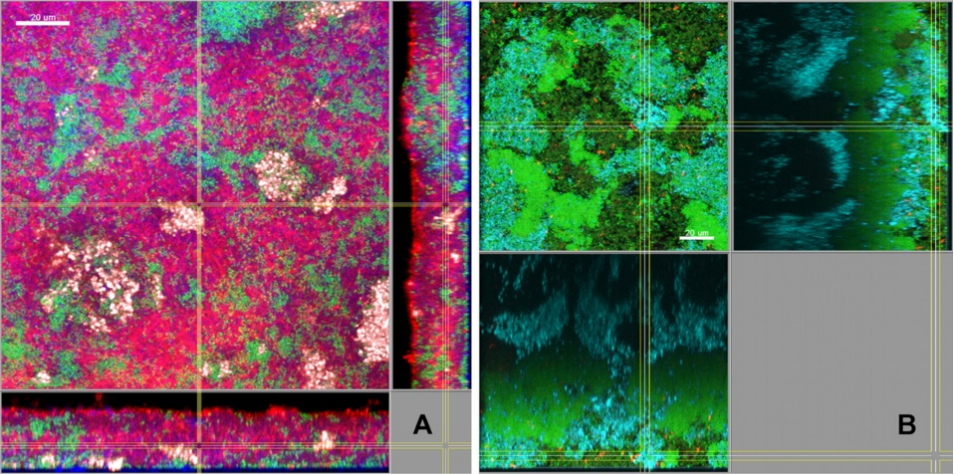
**Biofilms grown for 64.5 h in or mFUM4- (A) or iHS medium(B).** FISH staining of a fixed biofilm; the biofilm base in the side views is directed towards the top view. (**A**) red: *F. nucleatum*, white: *V. dispar*, green: non-hybridised cells, DNA staining (YoPro-1 + Sytox), blue: EPS. (**B**) cyan: streptococci, red: *F. nucleatum*, green: non-hybridised cells, DNA staining (YoPro-1 + Sytox). Figures show a representative area of one disc. Scale bars: 20 μm.

**Figure 6:**
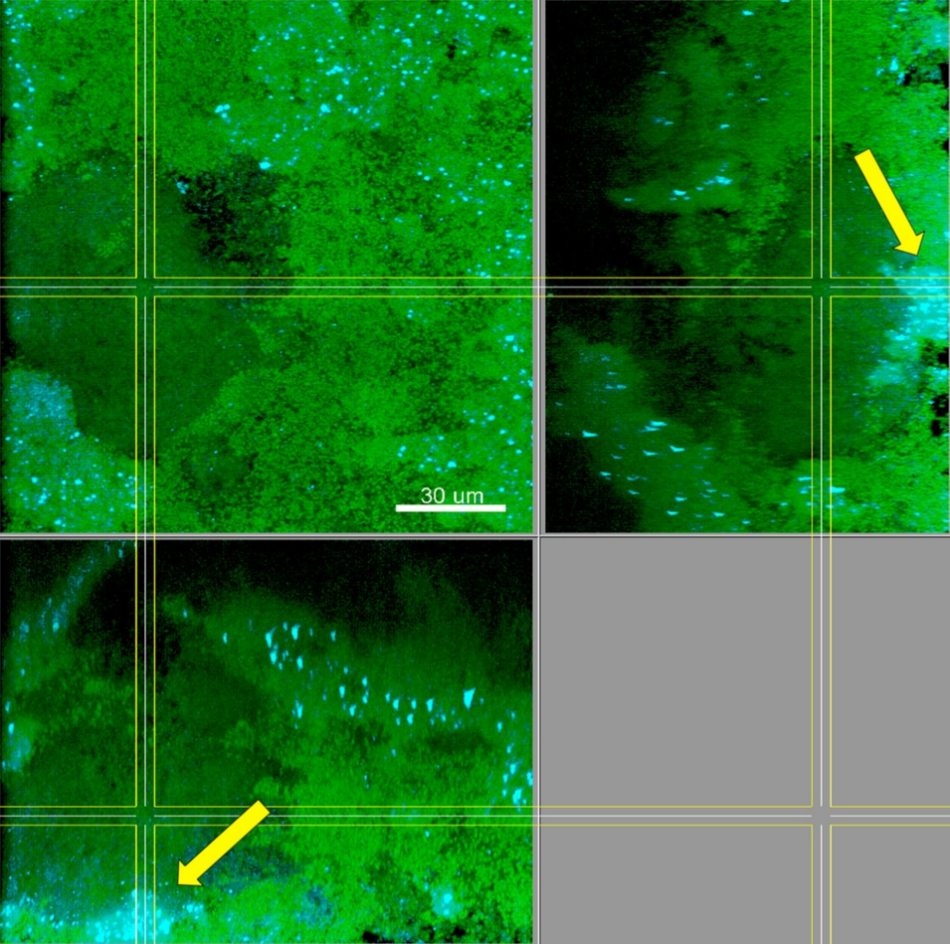
**Biofilms grown for 64.5 h in iHS medium.** FISH staining of a fixed biofilm; the biofilm base in the side views is directed towards the top view. Cyan: *V. dispar*, green: non-hybridised cells, DNA staining (YoPro-1 + Sytox). Arrows: Microcolonies of *V. dispar*. Shown is a representative area of one disc. Scale bar: 30 μm.

**Figure 7:**
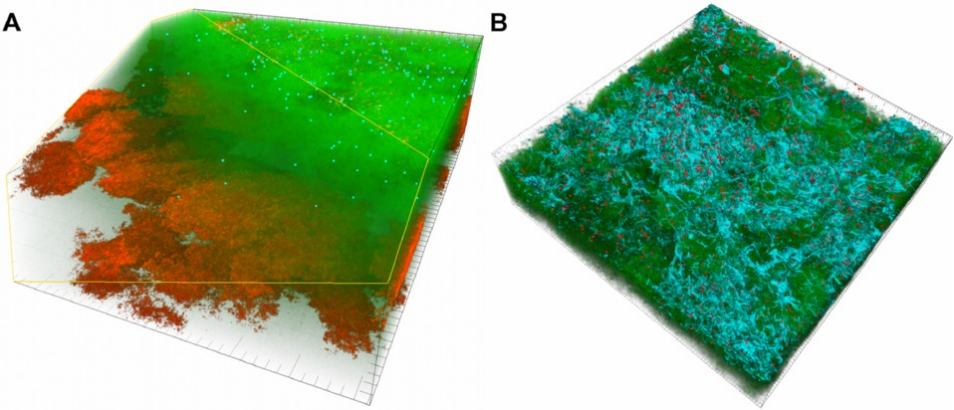
**3D-reconstructions of a 146 x 146 μm section of biofilms grown for 64.5 h in iHS medium.** FISH staining of a fixed biofilm. *P. gingivalis* and *T. forsythia* are shown schematically as dots (fluorescence maxima of the cells). (**A**) cyan: *T. forsythia*, red: *P. intermedia*, green: non-hybridised cells, DNA staining (YoPro-1 + Sytox). (**B**) cyan: *T. denticola*, red: *P. gingivalis*, green: non-hybridised cells, DNA staining (YoPro-1 + Sytox). Figures show a representative area of one disc.

**Figure 8:**
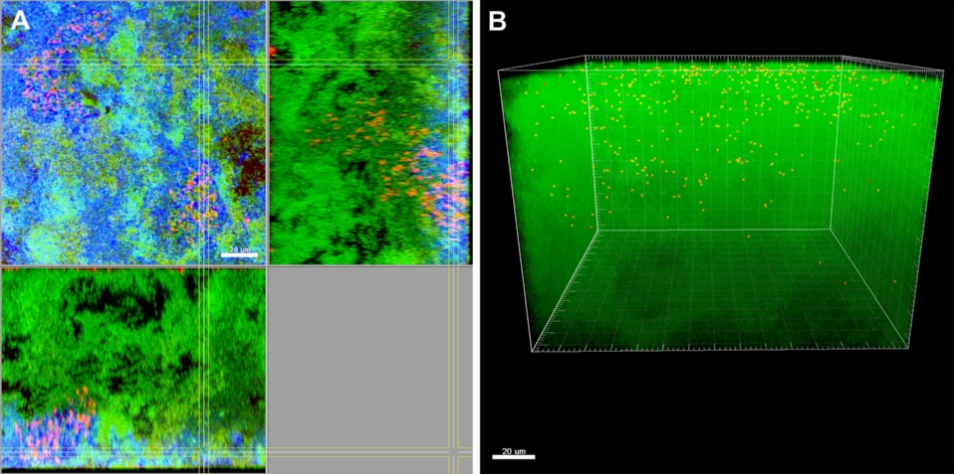
**Biofilms grown for 64.5 h in iHS Medium.** FISH staining of a fixed biofilm; the biofilm base in the side views is directed towards the top view. *C. rectus* is shown schematically as dots (fluorescence maxima of the cells). (**A**) red: *A. oris*, green: non-hybridised cells, DNA staining (YoPro-1 + Sytox), blue: EPS. (**B**) red: *C. rectus*, green: non-hybridised cells, DNA staining (YoPro-1 + Sytox). The red dots appear yellowish due to the transparency of the green channel. Figures show a representative area of one disc. Scale bars: 20 μm.

The quantitative representation of the used species was most convincing when biofilms were grown in iHS medium. *T. denticola* established in high numbers and the biofilms showed the best stability during the following staining procedures. Therefore, structural analysis was focused on these biofilms. CLSM analyses of FISH stained biofilms enabled us to determine all 10 species used in the model and locate their position in the biofilms. The top layer (approximately 30 μm from the biofilm surface) and basal layer (approximately 50 μm from the disc surface) of the biofilms showed clear structural differences and a fluent transition between these layers was observed. Biofilms grown in mFUM4 showed a dominance of *F. nucleatum* and streptococci in the basal layer (Figure 5A). In biofilms grown in iHS, however, *F. nucleatum* was detectable by FISH only in the top layer as dispersed cells, while streptococci were very abundant throughout the whole biofilm (Figure 5B). Aggregations of streptococci were often mixed with *V. dispar* in the whole biofilm except in the top layer, where *V. dispar* occurred as compact microcolonies (Figure 6). In biofilms grown in mFUM4, which had a lower thickness, this growth pattern of *V. dispar* was observed throughout the biofilm (Figure 5A). *P. intermedia* was found predominantly in the lower half of the biofilms forming microcolonies with diameters of about 50 μm on average (Figure 7A). *T. forsythia* was found mainly in the top layer of the biofilm, while none were detected in the lower half of the biofilms (Figure 7A). *T. denticola* grew loosely in the top layer alongside with *P. gingivalis,* which displayed the highest density in close proximity to *T. denticola* accumulations (Figure 7B). *A. oris* appeared as loose EPS-embedded microcolonies located in the upper half of the biofilms (Figure 8A). *Campylobacter rectus* was dispersed throughout the biofilm and did not form own microcolonies, but showed higher density in the top layer of the biofilm (Figure 8B).

## Discussion

This study focused on the importance of the nutritional conditions and the structure of subgingival biofilms generated on HA discs *in vitro.* The alteration of the growth medium by eliminating saliva and increasing the concentration of heat-inactivated human serum affected the biofilms positively as they developed to higher thickness, were more stable and enabled the extensive proliferation of *T. denticola,* which were observed only in small numbers using media with low or no heat-inactivated human serum. We were able to locate all the 10 organisms by multiplex FISH in combination with CLSM. The biofilms displayed a stratified structure reminiscent of *in vivo* subgingival biofilms [13]. However, in contrast to the *in vivo* situation, *F. nucleatum* was predominant in the basal layer along with streptococci of the biofilms grown in mFUM4. In biofilms cultured in iHS, *F. nucleatum* was detected as dispersed cells in the top layer. Earlier experiments showed that *F. nucleatum* has a strong dependency on streptococci, and is only able to establish along with them (data not shown). This observation is in accordance with the finding of co-aggregation studies that identified the ability of streptococci to attach to components of the pellicle, while *F. nucleatum* was shown to bind to the streptococci and act as a “bridging organism” for other species to colonize the biofilm [14]. The observed difference that *F. nucleatum* establishes in the basal layer might very well be due to the fact, that all strains were inoculated simultaneously. If no streptococci were added to the inoculum, but added to the biofilms at a later time point, *F. nucleatum* did not establish in the basal layer but rather after the addition of the streptococci, forming an intermediate layer. In this case, mainly *A. oris* was detected as an early colonizer (data not shown). Possibly, it would make sense to add the various strains sequentially, simulating the shift from health to disease.

The growth medium affected not only the biofilm composition; it had a strong influence on the rate of biofilm formation as well. The observed delay of biofilm formation in iHS medium could be explained possibly by the blocking of adhesion receptors on the saliva pellicle. It was shown for S*treptococcus pneumonia* and *Escherichia coli* that albumin inhibits biofilm formation on various surfaces [15,16]. It is very likely that this effect also occurs in our model during colonization of the discs. However, even though the initial attachment of the bacteria is prevented to a certain degree, all ten organisms were able to persist on the discs and were not washed away during dip-washing. Independent of the used medium, the biofilms showed a phase with a pronounced increase in thickness and bacterial abundance. This phase took about 20 h regardless of the used medium, however, the medium does affect its onset. Concluding this, it seems that a certain number of bacteria attached to the disc is required to promote “exponential” biofilm formation. Our experimental setup did not allow defining the reason(s) behind this phenomenon. Possibly, it is triggered by quorum sensing, as it was shown for several oral species that AI-2 or CSP signalling is involved in biofilm formation [17]. Alternatively, it could be that early biofilm formation under different nutritional conditions leads to different degrees of biofilm rigidity and therefore to different levels of sensitivity to shear-forces applied during biofilm dip-washing.

The iHS medium produced significantly higher cell numbers of *T. denticola* per biofilm compared to mFUM4 or SAL medium. However, *P. gingivalis* and *T. forsythia* were not affected by the higher serum concentration. This is surprising, since *P. gingivalis* was reported to profit from gingival crevicular fluid as well as from menaquinone secreted by veillonellae [17], and since one of the main growth factors of *T. forsythia,* N-acetyl-muramic acid [18], should be plenty available in thicker biofilms with probably increased proportions of lysing cells. On the other hand both species are known to be quite fastidious and our data indicate that it will be necessary to optimize further media components to increase their growth rates. *S. anginosus*, *A. oris*, and *V. dispar* showed mathematically significant reactions to the different growth media as well. However, in neither case the differences were greater than one log, which can hardly be considered as “biologically significant”.

The biofilms proliferating in iHS medium showed a consistent structure throughout the replicates and the organisms showed interactions as they could be expected according to literature. Zjinge et al. described three different layers in *in vivo* subgingival samples [13]. Our model biofilms showed differences between top- and basal layers as well, however, it was not possible to clearly define an intermediate layer. It rather seems that there is a fluent transition between top- and basal layer of the biofilms. The two layers show distinct characteristics. In the basal layer, biofilms were very compact and contained mainly streptococci, some veillonellae and large amounts of *F. nucleatum* and *P. intermedia*. The presence of *P. intermedia* was unexpected as it is in contrast to the *in vivo* situation where coccoid *Prevotella* species preferentially colonize the top layer in form of compact microcolonies [13]. The top layer of the model biofilms showed a rather loose structure with a lot of EPS. *V. dispar* and other cocci were embedded as compact microcolonies in their matrix, while *A. oris* appeared as loose microcolonies, with EPS surrounding each cell. In some preliminary diffusion experiments, similar to these described by Thunheer et al. for *in vitro* built supragingival biofilms [19], it seemed that these loose regions might work as diffusion channels, allowing large molecules to reach the basal layer in less than two minutes (data not shown).

The high abundance of *T. denticola* along with *P. gingivalis* and *T. forsythia* in the top layer was remarkable. The location, combined with the known high pathogenic potential of these species, might indicate a high inflammatory potential of our model biofilms. Particularly striking was to find *T. denticola* and *P. gingivalis* to colonize in close proximity, indicating some sort of metabolic dependency. This observation corresponds well with several previous studies. For example, it has been shown in a murine abscess model that the pathogenicity of *P. gingivalis* was significantly increased in presence of *T. denticola*[20]. The result was recently confirmed in a murine alveolar bone loss model, where co-inoculation showed a strong response not only for bone loss, but also for *P. gingivalis* specific T cell proliferation and interferon-γ production [21]. And in yet two other studies *P. gingivalis* and *T. denticola* had shown metabolic synergies by exchanging iso-butyric- and succinic acid [22] and an ability to co-aggregate with the Hgp44 domains of RgpA, Kgp and HagA acting as the key adhesins [23]. Other organisms found in this study in highest density in the top layer but without a specific focal distribution were *C. rectus*, *F. nucleatum* and *T. forsythia*. In the case of *C. rectus, a* highly motile microaerophilic organism, this meets the expectation. In biofilms grown in iHS medium, it was not possible to detect dense colonies of *F. nucleatum* in the basal layer by FISH, as it was the case in thin mFUM4 biofilms. There are several factors that could explain this finding. On the one hand, Sharma et al. made the same observation in two species biofilms of *F. nucleatum* and *T. forsythia*. Using a live-dead staining, they found mainly non-viable *F. nucleatum* attached to the substratum, while the bacteria in the upper layer of the biofilms showed a high viability [24]. Further, they observed synergistic growth of these organisms, which could explain the occurrence of *T. forsythia* together with the active *F. nucleatum* in the top layer of our biofilms. On the other hand, the effect could be explained by known problems of FISH staining procedure: The probes or the fixative possibly were not able to diffuse through the whole biofilm, or cells had strongly reduced number of ribosomes, leading to an extremely weak, non-detectable fluorescence. It is known that the obtained fluorescence intensity, with a few exceptions, is directly correlated with the growth rate of the target bacteria. The accessibility of the targets is controlled mainly by cell wall properties, which again require to get permeabilized by either the fixative or, in case of gram positive cells, lysozyme [25]. As *P. intermedia* and streptococci were readily stained at the base of the biofilms, a hindered diffusion of the probes or fixatives through the biofilms does not seem to be the problem. The accessibility of the cells can be sorted out as well, as the signal is very clear in the top layer of the biofilm. Careful examination of the images, by enhancing the contrast settings for the general DNA staining in our samples, revealed structures at the base of the biofilms that very much resembles the well-stained colonies of *F. nucleatum* observed in less thick biofilms. Combined with the high abundance detected by IF, it seems that *F. nucleatum* was in fact present in at the base of the biofilms, however, either in a non-viable- or at least non-active state. For future experiments, it might be worth investigating new methods to increase fluorescent signals, in order to obtain a bright staining throughout the whole biofilm. Catalysed reporter deposition (CARD)-FISH [26], the use of helper oligonucleotides [27], or designing probes targeting the 23S rRNA [28] might be solutions. Due to the large size of the horseradish peroxidase used with CARD-FISH, it seems unlikely that this method would be appropriate, and the use of helper oligonucleotides or probes targeting the 23S rRNA seem more promising to reach stronger signals.

One of the major differences to the *in vivo* situation is that the model biofilms grew without the presence of an epithelial cell layer. Some of the observed differences will be caused by the lack of interactions that occur *in vivo*. A future project will address this circumstance and aims to incorporate an epithelial cell layer into the model system. The main difficulty in maintaining such a co-culture system is that different growth conditions that are needed to cultivate either epithelial cells or biofilms. While the strict anaerobes in the consortium of the biofilms are very sensitive to oxygen, the epithelial cells do require oxygen for growth. Further, biofilms and epithelial cells do have very different nutritional requirements. In our co-culture experiments performed so far, cells and biofilms were cultured separately and incubated as co-culture after the development of both biofilms and epithelial cells [11]. Current experiments showed, that the biofilm consortium is still able to grow on agar plates after 48 h of co-culture, however, the viability of the bacteria was greatly reduced (data not shown).

## Conclusions

**Figure 9:**
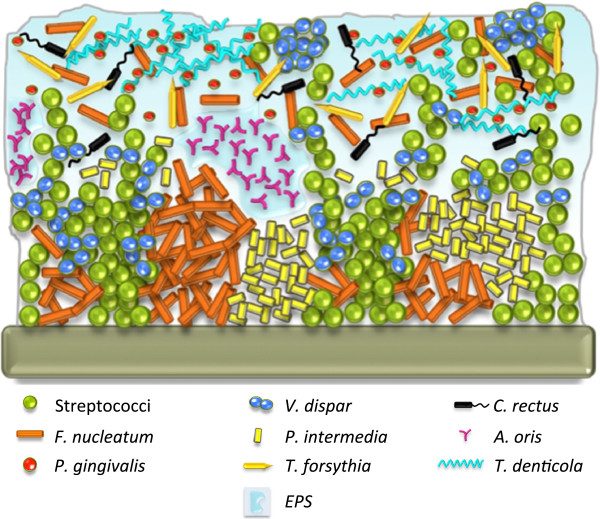
**Schematic structure of the 10-species *in vitro *biofilms after 64 h of incubation in iHS medium.** Distribution of the 10 species and EPS as observed by CLSM. The scale is not representative

We were able to locate all 10 species by CLSM and reconstruct the three-dimensional structure of our model biofilms (Figure 9). The structural analysis revealed a close proximity of *T. denticola* and *P. gingivalis* in the top layer of the biofilms, which might indicate a high pathogenic potential of these *in vitro* formed subgingival model biofilms. *V. dispar* appeared in the top layer as well, forming tight microcolonies.

The use of 50% heat-inactivated human serum in the growth medium improved the stability of the biofilms, resulting in significantly thicker biofilms. Under these conditions the fastidious *T. denticola* was able to establish in significantly higher densities compared to the media with 10% or no human serum. Surprisingly, neither *P. gingivalis* nor *T. forsythia* were affected by the concentration of human serum, and neither by the addition of saliva.

## Methods

### Biofilm generation and fixation

The biofilms used in this study are produced using a similar protocol as described before [11]. However, there are some key changes in the growth media and the strain composition that are described below. In the present study, *Streptococcus oralis* SK248 (OMZ 607), *Streptococcus anginosus* ATCC 9895 (OMZ 871), *Actinomyces oris* (OMZ 745; formerly *Actinomyces naeslundii*), *Fusobacterium nucleatum* subsp. *nucleatum* OMZ 598, *Veillonella dispar* ATCC 17748^T^ (OMZ 493), *Campylobacter rectus* OMZ 698, *Prevotella intermedia* ATCC 25611^T^ (OMZ 278), *Porphyromonas gingivalis* ATCC 33277^T^ (OMZ 925), *Tannerella forsythia* OMZ 1047, and *Treponema denticola* ATCC 35405^T^ (OMZ 661) were used.

All strains, except for *T. forsythia* and *C. rectus*, were maintained on Columbia blood agar (CBA). *T. forsythia* and *T. denticola* were maintained in liquid culture using the media outlined in Table 1. Prior to the onset of biofilm experiments, all strains were transferred into adequate liquid media (Table 1) for two cycles of precultures. The slow growing *T. forsythia*, *C. rectus* and *T. denticola* were precultured for 64 h (first cycle), then diluted 1:2 in fresh media and incubated for another 24 h (second cycle). All other strains were incubated over night (first cycle), diluted 1:10 in fresh media and incubated again for 8 h (second cycle). Prior to biofilm inoculation, all strains were adjusted to a defined optical density (OD_550_ = 1.0 except for *C. rectus*, *T. denticola* with OD_550_ = 0.5) and mixed in equal volumes. Sintered circular HA discs with a diameter of 10.6 mm (Clarkson Chromatography Products, South Williams-port, USA) were coated with 1:2 diluted saliva for pellicle formation. Discs were placed in 24-well polystyrene cell culture plates and covered with 1.5 ml of growth medium. In this study three different growth media, all based on mFUM [12], were used (Table 1). For inoculation 200 μl of the mixed precultures were added. Incubation was anaerobic and lasted 64.5 h. The medium was renewed after 16.5 h and subsequently every 24 h. After the first renewal of growth media, each well was supplemented with a boost of 40 μl of *T. denticola* liquid culture (OD_550_ = 0.5). Biofilms were dip-washed three times daily at intervals of 3–4 h. For dip-washings the discs were placed in 0.9% NaCl and washed by gentle agitation for 45 seconds. After this step, the discs were dipped twice two times each in two wells of fresh saline. Then the discs were returned to medium for further incubation.

**Table 1:** Growth media

**Medium**	**Abbreviation**	**Reference**	**Use**
mFUM, 4 mM Glucose	mFUM4		Growth medium for biofilms
mFUM 4 mM Glucose, iHS (50%)	iHS		Growth medium for biofilms
mFUM, 0.3% Glucose (30%), saliva (60%), iHS (10%)	SAL		Growth medium for biofilms
mFUM, 0.3% Glucose		[12]	Liquid precultures of *S. oralis*, *S. anginosus*, *V. dispar*^*1*^, *F. nucleatum*, *A. oris*, *P. intermedia*, *C. rectus*^*2*^
Pg medium^3^		[29]	Liquid precultures of *P. gingivalis*
Spirochaetes medium		[30]	Precultures of *T. denticola*
Modified OMIZ-W68^4^		[31]	Precultures of *T. forsythia*

^1^ addition of 1% lactic acid (v/v).

^2^ addition of 0.1% sodium fumarate and 0.1% sodium formiate.

^3^ Brain heart infusion broth, supplemented with haemin (7.67 μM) and menadione (2.91 μM).

^4^ addition of lactose (2 g l^-1^), caseinoglycomacropeptide (100 mg l^-1^),N-acetylmuramic acid (50 mg l^-1^), and N- acetylglucosamine (500 mg l^-1^).

For confocal microscopy, biofilms were fixed directly on the discs for at least 1 h at 4°C in 4% paraformaldehyde (Merck, Darmstadt, Germany) after the last dip-wash. For quantification by microscopic counting, biofilms were removed from the discs by vortexing (2 min in a 50 ml tube with 1 ml of in 0.9% NaCl) and sonicated for 5 sec at 25 W (Branson Sonic Power Company, Sonifier B-12) to reduce cell aggregation and the processed as described below.

### FISH staining procedure

The FISH procedure was done using the same conditions for the hybridisation as described by Thurnheer et al. [32]. Probe sequences, formamide concentrations used for the hybridisations, as well as the NaCl concentrations of the washing buffers are given in Table 2. To hybridise gram-positive bacteria, biofilms were pre-treated in lysozyme solution with a concentration of 1 mg/ml lysozyme (5 min, room temperature). The lysozyme solution consisted of 1 mg lysozyme from chicken egg white containing 70’000 units/mg (Fluka), dissolved in 890 μl H_2_O, 100 μl 1 M Tris–HCl solution (ICN Biomedicals, Inc.), pH =7.5, and 10 μl 0.5 M EDTA solution (Fluka), pH = 8.0. If the combination of probes required different formamide concentrations, the hybridisations were performed consecutively, starting with the highest concentration. Pre-hybridisation (15 min, 46°C) was performed in 500 μl hybridisation buffer without probes added. 500 μl of hybridisation buffer was used for each biofilm, supplemented with of up to 3 probes at a concentration of 10 ng/μl for each. The incubation time for the hybridisation was at least 3 h at 46°C in the dark. After the incubation, biofilms were transferred into washing buffer pre-heated to 48°C and incubated for 20 min at 48°C. For counterstaining, biofilms were stained using a mixture of 3 μM YoPro-1 iodide (Invitrogen) and 15 μM Sytox green (Invitrogen) (20 min, room temperature, in the dark) following the FISH procedure. To stain EPS, calcofluor (Sigma Chemical, Buchs, Switzerland); 10 μg/ml solution in 10 mM sodium phosphate, pH 7.5) was applied parallel to the counterstaining. After hybridisation the samples were embedded upside down on chamber slides in 100 μl of Mowiol [33].

**Table 2:** Sequences, labels and formamide concentrations for FISH Probes

**Organism**	**Name**	**Type**	**Labels**	**FA**^**1**^	**WB**^**2**^	**Sequence (5’ → 3’)**	**References**
*A. oris*	L-Act476-2	LNA^3^	Cy3, FAM, 6-Rox	40%	46 mM	ATCCAGCTACCGTCAACC	[11]
*C. rectus*	CAMP665	DNA	Cy3, Cy5	30%	112 mM	CATCTGCCTCTCCCTYAC	[11]
*F. nucleatum*	FUS664		Cy3, Cy5, FAM, FITC	40%	46 mM	CTTGTAGTTCCGCYTACCTC	[32]
	Fnuc133c	DNA	Cy3, Cy5	40%	46 mM	GTTGTCCCTANCTGTGAGGC	[11]
*P. intermedia*	L-Pint649-2	LNA	Cy3, FAM,6-Rox	40%	46 mM	CGTTGCGTGCACTCAAGTC	[11]
*P. gingivalis*	L-Pgin1006-2	LNA^3^	Cy3, Cy5, FAM	30%	112 mM	GTTTTCACCATCMGTCATC	[11]
Streptococci	STR405	DNA	Cy3, Cy5	20%	215 mM	TAGCCGTCCCTTTCTGGT	[34]
*T. denticola*	TrepG1_679	DNA	Cy3, Cy5, FAM	40%	46 mM	GATTCCACCCCTACACTT	[13]
*T. forsythia*	Tfor997	DNA	Cy3, Cy5, FAM	40%	46 mM	TCACTCTCCGTCGTCTAC	[35]
*V. dispar*	VEI217	DNA	Cy3, Cy5, FAM, FITC, 6-Rox	40%	46 mM	AATCCCCTCCTTCAGTGA	[32]

^1^ Formamide concentration in the hybridisation buffer.

^2^ Concentration of NaCl used in the washing buffer.

^3^ Probes containing locked nucleic acid substitutes (LNA). The ribose ring of LNA is constrained by a methylene linkage between the 2’ oxygen and the 4’ carbon.

### Quantification of FISH- and IF-stained bacteria

Harvested biofilms were quantified microscopically using FISH and IF. Samples were serially diluted, mounted and fixed on 24-well slides as described by Züger et al. [35]. *S. oralis*, *S. anginosus*, *T. denticola* and *V. dispar* were stained by FISH using the probes listed in Table 2, while *C. rectus*, *T. forsythia*, *P. gingivalis*, *P. intermedia*, *F. nucleatum* and *A. oris* were stained by IF using the monoclonal antibodies listed in Table 3. The protocols for FISH and IF, and the counting were as described by Züger et al. [35].

**Table 3:** Antibodies used for IF

**Target**	**Cell Line/MAb**	**Isotype**	**Reference**
*C. rectus*	212WR2	mouse IgG3	[36]
*T. forsythia*	103BF1.1	mouse IgG2b	[37]
*P. gingivalis*	61BG1.3	mouse IgG1	[38]
*P. intermedia*	37BI6.1	rat IgG2b	[39]
*F. nucleatum*	305FN1.2	mouse IgM	[40]
*A. oris*	396AN1	mouse IgM	[41]

### Structural analysis

Biofilms were stained directly on the hydroxyapatite (HA) discs by multiplex FISH and analysed by confocal laser scanning microscopy (CLSM) [32]. A Leica SP-5 microscope with resonant scanner system (8000 Hz), provided by the Centre of Microscopy and Image Analysis of the University of Zürich (ZMB), was used for imaging the biofilms. All images were captured using a 63x objective (glycerol immersion, NA 1.3). The system was equipped with a diode laser (405 nm excitation), an argon laser (458 nm/476 nm/488 nm/496 nm/514 nm excitation) and a helium neon laser (561 nm/594 nm/633 nm excitation). The laser settings varied depending on the used combination of probe labels (Cy3, Cy5, 6-Rox) and optimal settings were obtained using the spectra settings of the Leica software and/or the Invitrogen Fluorescence SpectraViewer (http://www.invitrogen.com/site/us/en/home/support/Research-Tools/Fluorescence-SpectraViewer.html) to adjust the settings manually. The thickness of the biofilms was determined using the xz view, and the measurement was performed using the measurement tool incorporated in the Leica software. For the creation of the stacked slice- and 3D - images, Imaris (Bitplane) was used.

### Statistical evaluation

All data presented in this study derive from three independent experiments. In each experiment, biofilms were cultured in triplicates for each examined time point and for each growth medium. Total counts presented in Figure 1 were determined by counting of colony forming units on CBA agar, while the total counts shown in Figure 3 were calculated based on the species-specific quantification by FISH and IF. One additional disc for each growth medium and time point was used to measure the thickness of the biofilms by CLSM. Using the logarithmized values of the abundances (N=9 values for each species), the Kruskal-Wallis test with p ≤ 0.05 was performed to determine the significance levels given in Figure 4. The thickness of the biofilms was measured on 9 independent biofilms, with N = 44 measurements on iHS biofilms, N = 61 on mFUM4 biofilms, and N = 57 on SAL biofilms. Significance was tested by ANOVA (Bonferroni test with p ≤ 0.001).

## Abbreviations

EPS: Exopolysaccharide; mFUM: Modified fluid universal medium; FISH: Fluorescence in situ hybridisation; IF: Immunofluorescence; CLSM: Confocal laser scanning microscopy.

## Authors' contributions

TWA designed the study, executed the experiments and drafted the manuscript. TT and RG supervised the study and edited the manuscript draft. All authors read and approved the final manuscript.
